# Cerebro-Spinal Meningitis

**Published:** 1872-09

**Authors:** J. H. Southall

**Affiliations:** Little Rock, Ark.


*Cerebro-Spinal Meningitis.* By J. H. SOUTHALL, M.D., Little Rock, Ark. (Read before the Pulaski County Medical Association, and published at the request of the Association.)

*Mr. President and Gentlemen of the Pulaski County Medical Association:*

It is with no false assurance of diffidence that I to-night, in accordance with the appointment of our president, submit for your consideration, and in furtherance of some discussion, as I understand its object, the subject of Cerebro-Spinal Meningitis; and in parenthesis will express the hope that the many imperfections discoverable, may have their "quantum sufficit" of allowance bestowed, because of your inexperienced narrator, and the brief space I propose to allot its consideration herein.

To begin, it is contended by many physicians that the name cerebro-spinal meningitis is not a very suitable one for this affection, being "too much of an anatomical bias, and an incomplete appreciation of facts." Notwithstanding, they have never rectified the name, except in applying such synonyms as petechial fever, spotted fever, malignant purpuric, fever febris, nigra-malignant meningitis, typhoid meningitis, etc., none of which seemingly express as much as the former, being certainly more destitute of the anatomical peculiarity, or proper appreciation of facts, inasmuch as all, or nearly so, are derivative from some one or more symptoms that are not at all times present in any single case. But, in fact, there is nothing in a name. Not one of us would treat disease according to what it might be styled, but address our remedies towards its cure through their application to the symptoms. The definition of cerebro-spinal meningitis, as adopted by Clymer, is that of an "acute specific disorder commonly happening as an epidemic, general or limited, and rarely sporadically; caused by some unknown external influence of sudden onset, rapid course, and very fatal. Its chief symptoms, referable to the cerebro-spinal axis, are, great prostration of the vital powers, severe pain in the head and along the spinal column, delirium, tetanic and sometimes clonic spasm, and cutaneous hyperæsthesia, and in some cases stupor, coma, and motor paralysis, attended frequently with hæmic spots. Its morbid anatomical characters being congestion and inflammation of the membranes of the brain and spinal cord, although there is reason to believe that the evidence of these changes may be wanting even in cases of long duration." Of its history little is known prior to the beginning of the nineteenth century, it having occurred probably both in this country and Europe without being recognized as a special disease of epidemic tendency until 1806, when it appeared at Medfield, Mass., as a distinct epidemic, and continued to appear as such through all the New England States until 1816. From this period until 1860 casual endemics, with sporadic cases here and there, have occurred through all the Southern and Western States; also from 1814 to 1857 circumscribed epidemics appeared in various parts of Europe.

Dr. Woodward, of the U. S. Army, and Dr. E. S. Gaillard, of the Confederate Army, both report the existence of the disease in their respective armies during the entire war, subsiding and reappearing at intervals; and many civilians fell victims to the disease in those cities South at which troops were stationed. In 1849 it appeared in Spain; from 1854 to 1861 in Sweden; in 1863 and 1864 in West Prussia; in 1866 in Dublin; and latterly it has prevailed in all the States of the Union, from Maine to Texas, and from the Carolinas to the Pacific; but its extensiveness among any population has not been in accordance with its wide geographical range; most generally confining itself to circumscribed localities within any one place.

The morbid anatomy of cerebro-spinal meningitis, as described by

Upham, Tourdes, Stillé, and Aitken, are opalescence of the upper surface of the cerebrum, vascularity of the membranes of the brain, chiefly of the pia mater, with a large increase of serum, mixed with flocculi of lymph, in the subarachnoid space and ventricles, with exudation of semi-coagulable lymph on base of brain, and medulla oblongata; a similar condition also exists in the spinal canal. These morbid appearances are found where death occurs a few days after inception of disease; when the disease has been of longer duration a cheese-like exudation is sometimes found, of some two or three lines in thickness, in close proximity to the meningeal vessels. In many of the cases reported the brain and spinal marrow have occasionally been implicated, the cerebrum having been found punctated and vascular, with both softening of it and cerebellum.

Mr. Rollet remarks, "that in those cases in which the substance of the brain is affected, there is a tendency to intermission, or at least remission, which alternates every three hours with an exacerbation;" this, though, he regards "as a characteristic symptom of the part affected, and not of malarial origin." In addition to the brain, spinal cord, and their membranes being the seat of disease, morbid changes from inflammation have been found in the alimentary canal, to which some pathologists have attached much importance, whilst others thought them merely accidental occurrences. Also the lungs, pleura, heart and its investments, have been involved during the progress of the disease. The heart has been found with its left side empty, and right filled with large fibrinous clots. "The blood during life, when drawn," according to Dr. Ames, of Alabama, "would form large loose coagula, in which all the red globules were rarely included, the serum separated slowly, and in small quantity; the color was in general bright; in a few cases approaching arterial blood," as per analysis of Mr. Tourdes. There was an increase of red globules and fibrin. The symptoms introductory to this disease are generally both formidable and sudden, being mostly ushered in by a severe chill followed by increased heat of surface, with pain in the head, extending down spine, with feelings of soreness or stiffness about the muscles of the neck, and again with heat of head, cold extremities, delirium, clonic spasm of limbs, flushed face, expressionless eye, with both dilated or contracted, or with one dilated, the opposite contracted, or occasionally the patient is attacked with double or triple vision, coma, more or less stupor, or again with pain in abdomen, accompanied by nausea and vomiting, cold bluish extremities, pulse thready. But whatever may be the initial symptoms, they are succeeded in a short time by clonic spasm or coma. I will just here narrate my observation of the symptoms of this disease as they appeared in two sporadic cases under my care in 1867 and 1868. Having kept but incomplete memoranda regarding them, I can of course give but a rough outline.

The first was that of a child two years of age, in whom the development of the disease was gradual, having its beginning in a chill accompanied with nausea and vomiting, and crying as if in pain, followed by feverishness, succeeded in twenty-four hours by delirium, restlessness, and clonic spasm of the extremities; body cold, head hot, tongue white but moist, pulse 130. This condition continued for four days, when the child manifested signs of improvement in a returning appetite, an abatement of the febrile symptoms, discontinuance of spasm, etc. Paralysis of left side now became apparent, notwithstanding which it continued to convalesce, and seemingly was getting well, until eight months thereafter, when it died from softening of the brain. The other case was that of a boy, aged twelve years. Whilst playing, he suddenly fell as if attacked by apoplexy, remaining quiet and comatose for half an hour, followed by a constant muttering, and clonic spasm of limbs, with occasional tetanic of opisthotonos variety; jaws locked; skin at periods both dry and moist; bowels bound; temperature of body from 99° to 103° F. There was no eruption. Patient died in thirty-six hours. Constipation, cephalalgia and rachialgia are almost constant symptoms, and require but a passing notice, being more or less severe in different cases. The pulse is but a poor guide in determining the nature or severity of an attack, being variable both in volume and frequency; but in the greater number of cases thready and weak, becoming intermittent in those assuming a fatal tendency. The temperature of the skin, as reported by Dr. Stille, varies between 100° and 105° F.

Dr. Charcot has made out the thermic periods for diseases of the cerebro-spinal axis, which, in my opinion, will be found to correspond more accurately with the facts in the case as the disease is presented to us this season. First, a rise to 100° F. from the normal standard. Second, oscillation between the normal temperature and 100° F. Third, a rapid rise to 105° F. and upwards, immediately before death.

As the eruption upon the body is by no means pathognomonic, and in fact I believe more frequently absent than present, I shall pay but little attention to *it*, merely mentioning that it may be from an ecchymosis to a pustular variety, and that when it does appear, it is generally within the first day or two. The duration of an attack may be from a few hours to several weeks, as deaths have been reported in as short a time as three hours. Should convalesence occur, the patient is susceptible of relapses, and many, painful and unpleasant sequelæ, of which paralysis, I believe, is the most serious. The mortality, as declared by Stille, Hirsch, and others, is from twenty-five to seventy-five per cent., the larger of which, I think, equals the percentage of losses sustained here, during its prevalence, since its inception at St. John's College in the latter part of the month of February. For instance, out of twenty-nine cases reported, we have had a fatal issue in twenty-one, with two cases undetermined (19th of April). The prognosis is therefore very unfavorable; especially is it so within the first five days.

As for the diagnosis of cerebro-spinal meningitis, the symptoms, even in the initial stage, all point to lesion of cerebro-spinal axis; so that in recollecting its physical diagnosis, we readily discover perversion of contractile discrimination, perversion of temperature, and structural perversion of the eye. Through this last much might be determined with the assistance of an opthalmoscope. Now, with derangement of one or more of these conditions always apparent in any single case, it seems quite improbable that any one with good discriminating judgment should confound it with any other complaint. It has been likened unto typhus fever, but its diagnostic differences are many, and would occupy too much of our space to point them out.

The cause is very plainly acknowledged by all as unknown, but presumed of course as some special disease agent, which does not convey contagion with its spread, possibly a something of a malarial character, as it is thought. Whatever it may be, it is of external influence; it is certainly neither heat nor cold, as the disease has appeared very nearly as often at one season of the year as at another.

In entering upon the treatment, it is with a feeling of regret and mortification that I have to assert its very unsatisfactory character. Many and numerous experiments have been made upon a scientific and empirical basis, without as yet evincing any special mode of treatment as preferable over another, and this I am fearful will continue to exist, until pathology, through the aid of analytical organic chemistry, discovers the morbid cause. But to the treatment: venesection is recomended in its beginning, where there is no prostration or coma. There is an article in the "Richmond and Louisville Medical Journal" for this month (April) where the author, in reporting a case, says he pushed the bleeding twice to syncope, removing from thirty-five to forty ounces at each sitting, and this at an interval of five hours. This case recovered (yet there were very few well-marked symptoms of the disease), and I presume in consequence he recommends the indiscriminate use of the lancet; but he assigns as a reason that the poison, whatever it may be, "acts as an irritant to the vaso-motor centre, producing contraction of the arteries and increased blood-pressure in the heart," and that the said blood-letting "relieves congestion of lungs, liver, stomach, and hyperæmic meninges, and lastly a threatening paralysis of heart." If his theory is correct, that the materies morbi acts directly upon the vaso-motor centres, bleeding is certainly indicated; but I will venture to assert that his theory is based upon an erroneous conclusion. The condition of the organs as described by him are but sometimes complications in the treatment of the disease, or found to exist post-mortem. And again, why should the disease be more confined to the motor than the sensory system, when we consider the part affected? The symptoms do not warrant us in the belief that one is more involved than the other, except in a few special cases. In fact, blood-letting as an indiscriminate remedial agent would, in my opinion, inflict more injury than its benefit in a few cases could counterbalance.

The treatment, according to my judgment, when reasoning upon hypothetical grounds as to cause and effect, to accomplish most, would be by whatever means possible to equalize the circulation, eliminate the poison from the blood, where presumption locates it, and to nourish and support the system whilst Nature performs the balance. The first and somewhat the second of these indications are met in the administration of hot hip-baths two or three times in the course of twenty-four hours, continued daily, and to be followed in two or more hours after each one, with as little exposure of person as practicable, with stimulating embrocations to trunk of body and limbs; next, to apply counter-irritants, such as emplast. cantharides, collod. cum cantharides, or comp. tinct. iodine, to back of head, neck, and down spine; opening bowels soon after first seeing patient with a stimulating enema of castor oil, oil of turpentine, and assafœtida, to be continued daily, as the necessity for keeping them open may demand. The medicine I should give internally would be bromide of potass., in combination with opiates, sufficiently to keep the patient under a sedative influence, and whatever other influence the bromide might exert.

According to Dr. J. H. Bill, United States Army, this sedative effect is brought about in a decrease of carbonic acid gas eliminated through the lungs, and which is still retained in the blood, it being really the sedative, and not the salt; and furthermore, that it is not a physical action, but a vital cause that acts through its effect upon nervous influence, limited to mucous membrane of the lungs. In other words, that "bromide of potassium, in its legitimate action, is an anæsthetic to the nerves of the mucous membranes, and a depressor of their action, and that hypnotic effects are secondary."

Dr. B. W. Richardson's experiments resulted in his promulgating the belief that "bromide and its salts might be considered a medicine, acting *primarily* upon the sympathetic or organic system of the nervous system," and as a modifier of vascular tension, and this whether applied locally and directly, or generally and indirectly. Now whether this sedative influence be appreciable through its action upon mucous membranes or attributable to its direct effect upon the brain, inasmuch as Dr. Bill further remarks, having partly proved by experiment and partly assumed its anæsthetic effect, when applied to the peripheral expansion of a nerve, says extend this inquiry: "If bromide changes cell nutrition, or produces anæsthesia in a nerve expansion, why not in ganglion, or ganglia? If it can prevent a nerve receiving or transmitting a sensation, why cannot it prevent the centres from appreciating and acting on the sensation; why cannot it reduce them to passivity—to sleep?" When we take these views of its sedative action in conjunction with its property of modifying vascular tension, lessening the active hyperæmia of the meningeal vessels resultant from an attack of this disease, do you not agree with me, that in combination with opium, which is claimed to possess so beneficial an influence, and promoting more cures than any other remedy, without any physiological reason to base its reputation upon, and only empiricism as a why or wherefore for its use, that probably the mortality in this dreadful disease might be lessened through its timely and judicious administration.

Dr. L. P. Yandell, Jr., mentions in an article upon the "Therapeutic Action of Bromide of Potassium," two sporadic cases cured by himself through its timely administration. He thinks the inefficiency of the drug in many cases is due to the insufficiency of the dose, or inadequate dilution by this last; he means, that instead of obtaining relief, you frequently induce great suffering through its irritant action on the stomach, as a small portion put upon the tongue and permitted to remain will induce soreness.

For the satisfaction of those gentlemen who may be advocates of the opium treatment exclusively, I will state that from experiments with the two combined, that rather than destroy the hypnotic or anodyne effect of the opiate, it heightens both, and more particularly the latter.

In pursuing the treatment to meet the third and last indication of support and nourishment of the system, it seems to me we should be very cautious in the use of stimulants, not though to their exclusion, but rely for the accomplishment of most good upon our dietary (milk and beef-tea). This last, if not tolerated by the stomach should be given by the rectum. If I thought stimulants were of use, I would prescribe egg-nogg, egg-flip, milk-punch, etc., etc.

And now, gentlemen, should my treatment have resulted in nothing more than the procurement of rest from struggling, and relief from pain, with time for Nature to endeavor to rid herself of the obnoxious cause, I have the satisfaction of knowing or believing I have mitigated the suffering of my patient, and given him the benefit of a somewhat scientific treatment.—*Richmond and Louisville Med. Jour.*

